# Detection of Residual Pluripotent Stem Cells in Differentiated Cells—General Requirements for Probe‐Based Quantitative PCR Assay

**DOI:** 10.1111/cpr.70276

**Published:** 2026-09-04

**Authors:** Jiani Cao, Ranran Xu, Boqiang Fu, Junying Yu, Tao Na, Aijin Ma, Jie Hao, Lei Wang, Hongling Zhao, Qiang Wei, Chunming Rao, Hao Wang, Zijiang Yang, Yanhui Zhang, Zuliang Zheng, Qiyuan Li, Weiqi Zhang, Qubo Chen, Shijun Hu, Lihua Zhou, Jing Fan, Siming Sun, Jian Xie, Cong Li, Tingting Zhang, Yidi Gan, Shuyan Wang, Glyn N. Stacey, Jun Wei, Tongbiao Zhao, Yu Alex Zhang

**Affiliations:** ^1^ Zephyrm Biotechnologies Co., Ltd. Beijing China; ^2^ Standard Committee, Chinese Society for Cell Biology Shanghai China; ^3^ National Institute of Metrology Beijing China; ^4^ Nuwacell Biotechnologies Co., Ltd. Hefei China; ^5^ National Institutes for Food and Drug Control Beijing China; ^6^ Beijing Technology and Business University Beijing China; ^7^ Beijing Institute for Stem Cell and Regenerative Medicine Beijing China; ^8^ National Stem Cell Resource Center, Institute of Zoology Chinese Academy of Sciences Beijing China; ^9^ University of Chinese Academy of Sciences Beijing China; ^10^ Institution of Laboratory Animal Science, Chinese Academy of Medical Sciences Peking Union Medical College Beijing China; ^11^ JOINN Pharmaceutical Quality Research and Testing (Beijing) Co., Ltd. Beijing China; ^12^ Betacure Therapeutics Co. Ltd. Jiaxing China; ^13^ Aperbio Technologies (Suzhou) Co., Ltd. Suzhou China; ^14^ Shenzhen Medical Academy of Research and Translation Shenzhen China; ^15^ China National Center for Bioinformation and Beijing Institute of Genomics Chinese Academy of Sciences Beijing China; ^16^ The Second Clinical College of Guangzhou University of Chinese Medicine Guangzhou China; ^17^ Soochow University Suzhou China; ^18^ Biology Division of National Institute of Measurement and Testing Technology Chengdu China; ^19^ Zhejiang Huode Bioengineering Co., Ltd. Hangzhou China

## Abstract

Residual PSC detection follows ultra‑trace‑analysis principles. LOD supports qualitative assessment. Assays employ PSC‑specific markers and proper references, with adaptation to differentiated cell populations from diverse PSC sources.

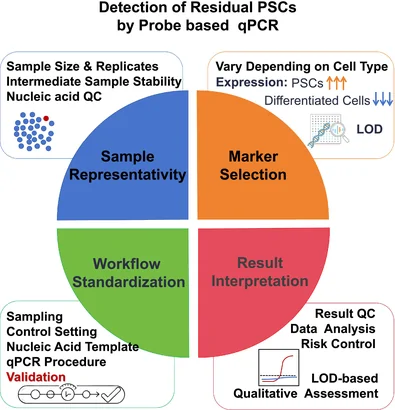

‘T/CSCB 0029‐2024 Detection of residual pluripotent stem cells in differentiated cells—General requirements for probe‐based quantitative PCR assay’ is jointly drafted and agreed upon by experts from the Chinese Society for Stem Cell Research. It applies to the development and validation of probe‐based real‐time quantitative PCR methods for detecting residual pluripotent stem cells (PSCs) in differentiated cell populations derived from PSCs. This document specifies the terminology, general requirements and procedures for the detection of residual PSCs in differentiated cells. It includes guidelines for cell sample collection and sampling volume, RNA extraction, reverse transcription, storage and stability validation of intermediate samples, selection of marker and reference genes, design of corresponding primers and probes, detection controls, quantitative PCR reaction programme design, result interpretation and validation requirements for the detection method. It was originally released by the China Society for Cell Biology on 28 October 2024. We hope that publication of the standard will facilitate standardization in assuring the safety of new PSC‐based therapeutics and support international efforts in therapeutic applications in stem cell and regenerative medicine.

## 1 Scope

This document specifies the terminology, general requirements and procedures for the detection of residual pluripotent stem cells (PSCs) in their differentiated cells. It includes guidelines for cell sample collection and sampling volume, RNA extraction, reverse transcription, storage and stability validation of intermediate samples, selection of marker and reference genes, design of corresponding primers and probes, detection controls, quantitative PCR reaction programme design, result interpretation and validation requirements for the detection method.

This standard is applicable to the development and validation of probe‐based real‐time quantitative PCR methods for detecting residual PSCs in their differentiated cell populations.

## 2 Normative References

The following documents contain provisions that are essential to the present document. Among these, the date of the referenced document is only applicable to the version indicated; the latest version (including all amendments) of the non‐dated document is applicable to the present document.

*GB/T 42077‐2022/ISO 20395:2019 Biotechnology—Requirements for evaluating the performance of quantification methods for nucleic acid target sequences—qPCR and dPCR*.

*GB/T 42076.1‐2022/ISO 20391‐1:2018 Biotechnology—Cell counting—Part 1: General guidance on cell counting methods*.

*GB/T 42753‐2023 General principles for performance evaluation of quantitative real‐time polymerase chain reaction analyser*.

*WS/T 230 Application of polymerase chain reaction (PCR) technology in clinical diagnosis*.

## 3 Terms and Definitions

The following terms and definitions apply to this document.

### 3.1 Differentiated Cell

A population of cells derived from stem cell differentiation, characterized by specific morphological structures and functional features.

### 3.2 Pluripotent Stem Cell

Stem cell that can self‐renew indefinitely in vitro and has the potential to differentiate into all three germ layers of cells.

Example: embryonic stem cells, induced pluripotent stem cells.

[SOURCE: GB/T42466‐2023, 3.2] [1].

### 3.3 Residual Pluripotent Stem Cell

Cells that remain undifferentiated during directed differentiation, retaining the characteristics of pluripotent stem cells and persisting within the differentiated cell population.

### 3.4 Negative Control Cell

Cell samples used as a negative reference, either primary somatic cells isolated from tissues of the same lineage as the test differentiated cells, or pre‐validated differentiated cell populations confirmed to be negative for residual pluripotent stem cells.

## 4 General Requirements

The development of methods for detecting residual pluripotent stem cells (PSCs) should adhere to the fundamental principles of trace or ultra‐trace target analysis. The limit of detection (LOD) is a critical parameter for qualitative assessment of results. Therefore, the selection of highly specific and abundantly expressed marker genes in PSCs, combined with stably expressed reference genes during differentiation, is essential to optimize LOD and ensure the accuracy of detection outcomes.

Given the variability in gene expression profiles among differentiated cell types derived from PSCs of different origins, method development should be tailored to specific functional cell populations differentiated from distinct PSC sources, following ICH Q2(R2) [2] and Q14 [3] guidelines.

This approach facilitates the identification of optimal marker and reference genes for each context.

To design analytical procedures for residual pluripotent stem cell detection, the following should be considered, including but not limited to:

- Sample collection and sampling volumes.
- RNA extraction and quality control.
- Reverse transcription and quality validation.
- Storage conditions and maximum allowable duration for intermediate samples, including cell lysates, RNA and cDNA.
- Marker gene/reference gene selection and primer/probe design.
- Implementation of detection controls.
- Design of qPCR procedure.
- Standardization of experimental workflows to minimize inter‐operator and inter‐batch variability.

## 5 Cell Sample Collection and Sampling Volume

### 5.1 Sampling

A minimum of three independent minimum packaging units shall be randomly selected from the same batch.

### 5.2 Sampling Volume

The amount of cells for testing shall be determined based on the following factors, including, but not limit to: therapeutic dosage specifications; minimum packaging unit specifications; LOD of the assay; cell number capacity of the RNA extraction method (i.e., ensuring the cell count is within the validated range for RNA isolation to maintain integrity and efficiency).

*Note:* In principle, a larger sample size increases detection sensitivity. Given that each test has a maximum sample volume limit, this can also be achieved by analysing multiple samples.

## 6 RNA Extraction

1. Purity and integrity of nucleic acid samples shall be considered following RNA extraction. RNA extraction protocols shall meet applicability requirements for cell sample processing (e.g., cell type compatibility, validated cell number range).

Note 1 For residual detection, RNA integrity is a critical factor influencing LOD and accuracy of results.

Note 2 Exceeding the validated cell number range of the RNA extraction method may compromise RNA integrity and purity.

2 The performance of the RNA purification method shall be evaluated during the analytical procedure development, which can be evaluated by a suitable technique, for example, nucleic spectrophotometry (see Annex A in Supporting Information), electrophoresis (see Annex B in Supporting Information), or Spike‐in qPCR with exogenous RNA controls (see Annex C in Supporting Information).
3 The influence of residual genomic DNA shall be fully considered during RNA extraction, and DNA elimination steps (e.g., DNase treatment, DNA‐binding columns) should be incorporated into the RNA extraction workflow. If primers are not designed to span introns or target intronic regions ≤ 500 bp in length, residual gDNA levels can be assessed using methods described in Annex D in Supporting Information.
4 RNA purity and concentration shall be determined, for example, using a microvolume UV spectrophotometer: Purity can be calculated as the OD260/OD280 ratio; Concentration can be calculated based on OD260 readings and appropriate dilution factors.
5 RNA samples should meet the following purity thresholds: 1.8 ≤ OD260/OD280 ≤ 2.2, and 1.8 ≤ OD260/OD230 ≤ 2.5.

## 7 Reverse Transcription

1. Reverse transcription methods and reagents shall be selected to promote the integrity of cDNA products, ensuring faithful representation of the original RNA template.
2. Reverse transcription efficiency shall be evaluated. An example of the methodology for detecting reverse transcription efficiency can be referred to Annex E in Supporting Information.
3. Total RNA input per reaction should be ≥ 1 μg and shall adhere to the manufacturer's specified input range for the reverse transcription kit.
4. Dilution of cDNA for downstream detection should follow the following principles:
  - No dilution is required if the Ct value of the undiluted cDNA reference gene falls within the quantitative linear range of the assay.
  - Dilution is necessary if the Ct value of the undiluted reference gene is below the lower limit of the quantitative linear range.
  - Minimize dilution factors to preserve low‐abundance targets critical for residual detection.
  - The linear range of reference gene detection should be validated according to the technical requirements in 8.5 of GB/T 42077/ISO 20359.

*Note:* Excessive dilution may compromise the limit of detection (LOD) for residual targets due to reduced template availability.

## 8 Storage and Stability Validation of Intermediate Samples

1. Intermediate samples (e.g., cell lysates, RNA, cDNA) should be processed immediately to minimize the risk of false‐negative results due to degradation of low‐abundance residual targets.
2. If storage of intermediate samples is necessary, conditions and durations shall be validated under LOD conditions for residual PSC detection. Recommended storage conditions and validation methods for different intermediate samples are provided in Annex F in Supporting Information.
3. Intermediate samples should not undergo repeated freeze–thaw cycles to preserve target stability and integrity.

## 9 Marker/Reference Gene Selection and Primers/Probe Design

### 9.1 Marker Gene Selection

#### 9.1.1 Selection Principles

- Marker genes shall exhibit specific and stable expression in PSCs and no expression in the differentiated cell population under test.
*Note:* It is imperative to account for the variability among distinct undifferentiated PSC populations and their respective expression levels.
- When multiple candidate genes meet the above criteria, priority should be given to genes that are consistently negative across a wide range of differentiated cell types.

#### 9.1.2 Screening Methods

Preliminary screening of marker genes for residual PSC detection can be performed using high‐throughput transcriptomic analysis comparing PSCs and their differentiated derivatives. A list of publicly reported marker genes for residual PSC detection is provided in Annex G in Supporting Information.

#### 9.1.3 Validation of Marker Genes

Candidate marker genes for residual PSC detection shall satisfy the following validation criteria:

- Primary somatic cells isolated from tissues of the same lineage as the test cell product should be used as negative controls for marker gene validation.
- If the differentiated cells have to be used as negative controls, the batch used shall be pre‐validated as PSC‐free through a suitable alternative validated assay, for example, in vivo teratoma assays and in vitro pluripotency culture tests.
- The expression of the marker gene in negative control cells shall be undetectable (no typical amplification curve) or ultra low (Ct value ≥ 35).
- Handling Background Amplification: If weak amplification (Ct ≥ 35) with specific curves is observed in negative controls, establish a baseline Ct value (Ct_baseline_) by testing ≥ 3 independent batches of pre‐validated negative control cells under standardized conditions (sampling volume, RNA extraction/reverse transcription protocols, template input, qPCR parameters).
- A candidate gene shall not be used as the marker for residual PSC detection if Ct_baseline_ < 35 in negative controls.
- The marker gene shall demonstrate stable and robust expression in PSCs, with qPCR results showing consistent amplification curves and Ct values ≤ 25 under validated assay conditions.

### 9.2 Reference Gene Selection

#### 9.2.1 Stability Criteria

Reference genes shall be selected based on their stable expression, exhibiting minimal change throughout differentiation.

#### 9.2.2 Ct Value Range

Reference genes should exhibit Ct values within the range of 10–25 under defined assay conditions (including cell count per sample, nucleic acid extraction, reverse transcription and the qPCR system).

### 9.3 Primer and Probe Design

1. Primers and probes targeting mRNA sequences of marker genes and reference genes shall comply with the design criteria and amplicon length requirements specified in GB/T 42077/ISO 20395, section 6.1. Intron‐spanning design should be preferential to eliminate false‐positive amplification from residual genomic DNA.
2. Multiplex qPCR utilizing marker and reference gene probes labelled with distinct fluorophores can be applied.

*Note:* Due to spectral overlap between fluorescence channels, fluorophores with adequate emission wavelength separation should be prioritized to minimize cross‐talk.

## 10 Design of qPCR Procedure

### 10.1 Amplification Strategy

One‐Step RT‐qPCR (direct amplification using RNA templates) or Two‐Step RT‐qPCR (amplification using cDNA templates synthesized in a separate reverse transcription step) or an equivalent validated technique can be applied to PSC detection, following manufacturer‐recommended master mix formulations and cycling protocols.

### 10.2 Real‐Time qPCR Setup

#### 10.2.1 Reaction Assembly

Reactions should be prepared according to the qPCR kit manufacturer's instructions with the following specifications:

Minimum reaction volume: ≥ 20 μL per tube.

Template input: Maximize template volume within the validated linear range.

Replicates: ≥ 3 technical replicates per test sample.

#### 10.2.2 Thermal Cycling Parameters

Real‐time PCR instruments shall comply with GB/T 42753 specifications and include fluorescence detection channels compatible with the selected probe reporter dyes.

Thermal cycling parameters settings should adapt instrument model and reagent specifications. Total cycles should be ≥ 40 cycles. The threshold value shall be set according to the requirements in GB/T 42077/ISO 20395, section 7.3.1.

## 11 Limit of Detection (LOD)

1. The LOD should be established using a spike‐in assay, where PSCs are serially diluted into negative control cells at defined ratios (see Annex H in Supporting Information).
2. LOD Validation should meet the following requirements:
  - If the marker gene is undetectable (no amplification curve) in negative control cells: The LOD can be defined as the lowest spike‐in ratio where the marker gene is consistently detected in all replicates across multiple batches.
  - If the marker gene exhibits background amplification (Ct ≥ 35) in negative control cells: The LOD can be defined as the lowest spike‐in ratio where: the marker gene is consistently detected in all replicates; and the ∆∆Ct between the lowest detectable spike‐in sample and the highest‐expressing negative control sample is ≤ −1.
3. When reporting ‘undetectable’ results, the LOD of the method shall be explicitly stated.

## 12 Control Setup

Controls shall be included to assess the overall quality and reliability of the generated data. These include, but are not limited to:

- Positive control: A positive control reaction is performed using nucleic acid template containing the marker sequence (e.g., plasmid DNA containing the marker sequence, RNA or cDNA derived from pluripotent stem cells). The template input shall yield consistent detection with Ct values ≤ 25 under validated qPCR conditions.
- Negative template control: A reaction is performed using sterile water or buffer solution instead of nucleic acid template.
- Reverse Transcription Negative Control: If the primers and probes are located in the same exon region or the length of the introns crossed is small (less than or equal to 500 bp), a control reaction should be performed without reverse transcriptase to assess genomic DNA contamination.
- Cell negative control: Pre‐validated cells confirmed to lack marker gene expression (e.g., primary somatic cells of the same lineage as the test cells or other somatic cells with confirmed negative expression) treated and analysed in parallel with test samples to control for background signals.

## 13 Results Interpretation

### 13.1 Quality Control

The following conditions shall be met for the experimental results to be valid:

- Positive Control: Ct value: ≤ 25.
- Negative Template Control: Undetectable (no amplification curve) or the minimum Ct value ≥ 35 (if background amplification is observed).
- Reverse Transcription Negative Control: Undetectable (no amplification curve) or the minimum Ct value ≥ 35.
- Cell Negative Control: Undetectable (no amplification curve) or the minimum Ct value ≥ 35.
- Reference Gene in Test Samples: Ct value: 10 ≤ Ct ≤ 25.

### 13.2 Result Interpretation

Qualitative interpretation of results shall adhere to the following criteria.

#### 13.2.1 Negative Result

If no Ct value or specific amplification curve is observed for the marker gene in the test sample, or the minimum Ct value of the marker gene in the test sample (Ct_sample,min_) is ≥ Ct_baseline_, it is concluded that residual PSCs are undetectable, with levels below the limit of detection (LOD).

#### 13.2.2 Positive Result

If the maximum Ct value of the marker gene in the test sample (Ct_sample,max_) is < Ct_baseline_ and a specific amplification curve (e.g., sigmoidal shape) is observed, conclude the residual PSCs are detected, with levels above the LOD.

## 14 Method Validation

1. Validation shall be performed prior to implementing the residual PSC detection method. The requirements in GB/T 42077/ISO 20395, section 8 should be followed, method performance parameters include but are not limited to:
  - Specificity.
  - Precision.
  - Linear range.
  - Limit of Quantitation (LOQ).
  - Limit of Detection (LOD).
  - Trueness.
  - Robustness.
2. Critical factors affecting result interpretation or LOD drift shall be fully considered, including but not limited to:
  - Lineage and differentiation status of test cell products.
  - RNA extraction and reverse transcription protocols.
  - Primer/probe sequences and concentrations.
  - PCR master mix composition.
  - qPCR instrument parameters.

If any of the above factors are modified, method revalidation shall be performed according to the requirements in GB/T 42077/ISO 20395, section 8.

## Author Contributions

Jiani Cao, Jun Wei, Tongbiao Zhao and Yu Alex Zhang contributed to conception and design. Jiani Cao, Ranran Xu, Boqiang Fu, Junying Yu, Tao Na, Aijin Ma, Jie Hao, Lei Wang and Hongling Zhao drafted and revised the manuscript. Qiang Wei, Chunming Rao, Hao Wang, Zijiang Yang, Yanhui Zhang, Zuliang Zheng, Qiyuan Li, Weiqi Zhang, Qubo Chen, Shijun Hu, Lihua Zhou, Jing Fan, Siming Sun, Jian Xie, Cong Li, Tingting Zhang, Yidi Gan, Shuyan Wang, Glyn N. Stacey, Jun Wei, Tongbiao Zhao and Yu Alex Zhang critically read and revised the manuscript.

## Funding

This work was supported by the National Key Research and Development Program of China, 2023YFC3605100; Chongqing Key Technology Innovation and Application Program, CSTB2025TIAD‐STX0044.

## Supporting information


**Data S1:** Annexes A–H.

## Data Availability

Data sharing not applicable to this article as no datasets were generated or analysed during the current study.
