# Supplementary material for: Detection of Residual Pluripotent Stem Cells in Differentiated Cells—General Requirements for Probe‐Based Quantitative PCR Assay
Source: Cell Prolif. 2026 Sep 4:e70276. Online ahead of print. doi: 10.1111/cpr.70276 (PMC13545406; doi:10.1111/cpr.70276)
Supplement: Supplementary file 1 [file CPR-9999-e70276-s001.docx]

**Annex A**

**(Informative)**

**Performance Evaluation of RNA Extraction Methods - Nucleic Acid Yield and Purity: Spectrophotometry**

**A.1 General**

The performance of RNA extraction methods is evaluated based on RNA yield per cell and purity metrics. Use a validated cell counting method to determine the exact cell number for each sample. Perform RNA extraction using the method(s) under evaluation, include ≥ 6 technical replicates per extraction method to ensure statistical robustness. Measure RNA concentration and purity using a UV spectrophotometer. Calculate RNA yield per cell. Perform statistical analysis on RNA yield and purity data. Compare extraction methods based on yield consistency and purity thresholds.

**A.2 Experiment Design**

**Sample Preparation:** Prepare test cell suspensions at an **optimized density** (Con_cell_) using validated cell counting methods. Aliquot identical volumes (V_cell_) of the suspension into labeled microcentrifuge tubes. Centrifuge to obtain cell pellets.

**Group Assignment:** Randomize cell pellet samples into experimental groups for parallel evaluation of RNA extraction methods (Method A, B, C, etc.). Each method group must include **≥6 technical replicates** (N_sample_).

**RNA Extraction:** Perform RNA extraction according to manufacturer protocols. Quantify RNA using a microvolume spectrophotometer: **RNA concentration** (Con_RNA_, ng/μL) ; **Purity ratios** (OD_260_/OD_280_ and OD_260_/OD_230_) and Record RNA volume (V_RNA_, μL).

**Purity Evaluation:** Count samples failing purity thresholds:

N_OD260/280_: Samples with OD_260_/OD_280_ ∉ [1.8, 2.2]

N_OD260/230_: Samples with OD_260_/OD_230_ ∉ [1.8, 2.5]

Calculate non-pure extraction probability (P_np_):

P_np_(%)=(N_OD260/OD280_ + N_OD260/OD230_) / (2 × N_sample_) × 100%

Higher P_np_ indicates poorer method purity performance.

**Yield Evaluation:** Calculate **RNA yield per cell** (Pro, ng/cell):

Pro = Con_RNA_ × V_RNA_ / (Con_cell_ × V_cell_)

Higher Pro indicates superior RNA recovery efficiency.

**Annex B**

**(Informative)**

**Performance Evaluation of RNA Extraction Methods - Integrity: Electrophoresis**

**B.1 General**

RNA integrity can be assessed using electrophoretic profiles presenting distinct for different molecule groups ribosomal RNA. The electrophoretic profile of mammalian RNA typically exhibits three discernible band corresponding to the 28S, 18S, and 5S ribosomal subunits. Under optimal conditions, the intensity of the 28S band is approximately twice that of the 18S band. Partial degradation of RNA is indicated by the presence of blurred or diffuse bands. The absence of distinct bands or the presence of only low molecular weight species signifies complete RNA degradation, necessitating re-extraction.

**Annex C**

**(Informative)**

**Performance Evaluation of RNA Extraction Methods- Integrity: Spike-in qPCR**

**C.1 General**

A defined quantity of exogenous RNA containing a non-expressed target sequence (specific to the test sample) is spiked into a controlled number of biological samples. Parallel aliquots are subjected to RNA extraction using different methodologies under evaluation, with a minimum of six replicates per method. The extracted RNA is analyzed using a validated one-step qPCR protocol to quantify the spiked RNA. Extraction efficiency can be evaluated based on Ct values obtained from spiking qPCR assay.

**C.2 Experimental Design Example**

**Sample Preparation:** Prepare cell suspensions of appropriate concentration from test samples based on predetermined cell counts. Aliquot uniform volumes into sufficient 1.5 mL microcentrifuge tubes. Centrifuge to pellet cells and discard supernatant. Spike each pellet with an equal quantity of RNA reference material (RNAref) containing the non-expressed target sequence.

**Group Assignment:** Randomize spiked samples into experimental groups for different RNA extraction methods (Method A, B, C, etc.), each containing ≥ 6 replicates. Process immediately for RNA extraction.

**RNA Extraction:** Perform RNA extraction according to protocol specifications for each method. Normalize the volume of the extracted RNA to a defined quantity and recorded as V_RNA._ Quantify RNA concentration and purity via spectrophotometric quantification.

**Positive Reference (REF):** Dilute equal quantity of RNAref to match the final volume (V_RNA_) of extracted RNA samples.

**qPCR Analysis:** Quantify the spiked RNA in each sample by one-step qPCR. Include at least three technical replicates per sample. Record Ct values for both test samples (Ct_target_) and positive reference (Ct_ref_). Calculate mean Ct_target_ and Ct_ref_ values per experimental group.

Performance Evaluation: Lower Ct_target_ values, particularly those approaching Ct_ref_, indicate higher RNA integrity.

**Annex D**

**(Informative)**

**Evaluation of Residual Genomic DNA**

**D.1 General**

Conserved non-transcribed genomic sequences are selected as the detection targets. One-step qPCR protocol is used to quantify the target sequence in test samples. Residual genomic DNA content is assessed based on the cycle threshold (Ct) values obtained from target amplification.

**D.2 Experimental Design Example**

**Sample Preparation:** Collect an adequate quantity of test sample cells according to experimental requirements. Prepare cell suspensions at appropriate concentrations based on cell counting. Aliquot uniform volumes into a sufficient number of 1.5 mL microcentrifuge tubes as required for the assay. Centrifuge to pellet cells and discard supernatant, generating parallel cell pellet replicates.

**Grouping:** Randomize samples into experimental groups for RNA extraction and genomic DNA removal treatment (Method A, Method B, Method C...), each group containing ≥ 6 replicates.

**gDNA Control: Genomic DNA from the same type of cell sample.**

**Quantitative PCR (qPCR) Detection:** Perform target sequence detection on test samples and gDNA controls using a validated one-step qPCR protocol. Apply the maximum recommended template input and include at least three technical replicates per sample. Determine the target sequence Ct values (Ct_target_) and their respective mean Ct values for each group.

**Performance Evaluation:** Provided that the gDNA control shows positive amplification, a negative result for the target sequence in the test sample indicates excellent removal of genomic DNA. Lower Ct_target_ values indicate poorer removal efficiency of genomic DNA. If Ct_target_ < 35, the corresponding genomic DNA removal method is not recommended for detecting residual pluripotent stem cells.

**Annex E**

**(Informative)**

**Evaluation of Reverse Transcription Efficiency**

**E.1 General**

Prepare reverse-transcribed cDNA (R-cDNA) samples from a gradient of RNA concentrations and a corresponding gradient of diluted control cDNA (C-cDNA) samples. Quantify a suitable detection target sequence (a stably and positively expressed sequence in the cell sample) using a validated qPCR protocol. Assess reverse transcription efficiency based on the Ct values obtained from target amplification.

**E.2 Experimental Design Example**

R-**cDNA Sample Preparation:** Perform reverse transcription using a gradient of RNA quantities (e.g., 1 μg, 500 ng, 250 ng, 125 ng, 62.5 ng) to generate a series of cDNA samples, designated as R-cDNA.
C-**cDNA Sample Preparation:** Take 1 μg of reverse-transcribed cDNA and perform a 2-fold serial dilution, following the same dilution scheme as the RNA gradient. Designate these as C-cDNA samples.

**Quantitative PCR (qPCR) Detection:** Select a suitable detection target which is stably and positively expressed in the cell sample. Quantify the target sequence for both R-cDNA and C-cDNA samples using a validated qPCR protocol. Record the Ct values for each sample.

**Performance Evaluation:** Generate standard curves for both the R-cDNA gradient samples and the C-cDNA gradient samples based on the qPCR results. Evaluate reverse transcription efficiency by comparing the degree of overlap between the two standard curves. Reverse transcription efficiency is positively correlated with the degree of overlap between the curves.

**Annex F**

**(Informative)**

**Validation of Storage Conditions and Shelf Life for Intermediate Samples**

**F.1 General**

Prepare parallel intermediate LOD test samples (e.g., cell pellets, cell lysates, RNA, cDNA). Subject the samples to different storage conditions and gradient storage durations. At designated time points, retrieve experimental group samples for subsequent testing. Validate the storage conditions and shelf life for experimental groups where all LOD samples yield positive results.

**F.2 Reference Storage Conditions**

Recommended storage conditions for intermediate samples are shown in Table 1.

**Table 1: Reference Storage Conditions for Intermediate Samples**

| **Sample Type** | **Storage Conditions** |
| --- | --- |
| Cell pellets | Short-term storage: 2℃–8℃; Long-term storage: -80℃ |
| Cell Lysate | Short-term storage: 2℃–8℃; Long-term storage: -80℃ |
| RNA | Short-term storage: 2℃–8℃; Long-term storage: -80℃ |
| cDNA | Short-term storage: 2℃–8℃; Long-term storage: -20℃ or -80℃ |

**F.3 Validation Sample Preparation and Experimental Design**

**F.3.1 Validation Sample Preparation**

1. **Negative Control Cell Samples and LOD Samples:**

a. Prepare an adequate quantity of negative control cell samples according to experimental requirements. Adjust the cell suspension to an appropriate concentration based on cell counting.

b. Aliquot the suspension into 1.5 mL microcentrifuge tubes according to the cell number specified in the testing protocol. Use these aliquots to prepare negative control cell samples and LOD spiked samples as described below.

c. **Negative Control Cell Samples:** Centrifuge the aliquots from step b, discard the supernatant, and obtain parallel cell pellet replicates.

d. **LOD Samples:** Spike the aliquots from step b with a defined number of pluripotent stem cells at the LOD level. Centrifuge, discard the supernatant, and obtain parallel cell pellet replicates.

2. **Positive Control Samples:**

Prepare pluripotent stem cell samples as positive controls.

**F.3.2 Validation Experimental Design and Acceptance Criteria**

Refer to Table 2 for an example of the validation protocol and corresponding acceptance criteria for intermediate sample shelf life.

**Table 2: Validation Protocol for Intermediate Sample Shelf Life**

| **Sample Type** | **Experimental Design** | **Acceptance Criteria** |
| --- | --- | --- |
| Cell pellets/Cell Lysate | **Parallel Preparation of Samples:** Prepare an adequate quantity of negative control cell samples, limit of detection (LOD) samples, and positive control samples in parallel according to experimental requirements.  **Storage Conditions and Time Points:**  **2°C–8°C Storage:** Design groups with gradient time points (e.g., immediate testing, 1 hour, 2 hours, 4 hours).  **-80°C Storage:** Design groups with gradient time points (e.g., immediate testing, 3 days, 5 days, 10 days).  Include ≥3 replicates per sample type for each group.  **Testing Protocol:** Perform immediate testing on one group of samples as a control, store the remaining samples according to the experimental design (At 2°C–8°C in a refrigerator or at -80°C in a freezer). Retrieve samples at the designated time points for subsequent testing. Evaluate results based on the parameters specified in the detection procedure. | An experimental group is considered validated if the following conditions are met:   1. All negative control cell samples in the experimental group are determined to be negative. 2. All LOD samples in the experimental group are determined to be positive. 3. All positive control samples in the experimental group are determined to be positive. |
| RNA | **Parallel Preparation of Samples:** Prepare an adequate quantity of negative control cell samples, limit of detection (LOD) samples, and positive control samples in parallel according to experimental requirements. Perform RNA extraction for all samples in a single experiment.  **RNA Aliquot Preparation:** Aliquot the extracted RNA into equal volumes based on the template amount required for the experimental design.  **Storage Conditions and Time Points:**  **2°C–8°C Storage:** Design groups with gradient time points (e.g., immediate testing, 1 hour, 2 hours, 4 hours).  **-80°C Storage:** Design groups with gradient time points (e.g., immediate testing, 3 days, 5 days, 10 days).  Include ≥3 replicates per sample type for each group.  **Testing Protocol:** Perform immediate testing on one group of samples as a control, store the remaining samples according to the experimental design (At 2°C–8°C in a refrigerator or at -80°C in a freezer). Retrieve samples at the designated time points for subsequent testing. Evaluate results based on the parameters specified in the detection procedure. |  |
| cDNA | **Parallel Preparation of Samples:** Prepare an adequate quantity of negative control cell samples, limit of detection (LOD) samples, and positive control samples in parallel according to experimental requirements. Perform reverse transcription for all samples in a single experiment.  **cDNA Aliquot Preparation:** Aliquot the **cDNA** into equal volumes based on the template amount required for the experimental design.  **Storage Conditions and Time Points:**  **2°C–8°C Storage:** Design groups with gradient time points (e.g., immediate testing, 4 hour, 24 hours, 48 hours).  **-20°C Storage:** Design groups with gradient time points (e.g., immediate testing, 3 days, 10 days, 30 days).  **-80°C Storage:** Design groups with gradient time points (e.g., immediate testing, 3 days, 30 days, 60 days).  Include ≥3 replicates per sample type for each group.  **Testing Protocol:** Perform immediate testing on one group of samples as a control, store the remaining samples according to the experimental design (At 2°C–8°C in a refrigerator or at -80°C in a freezer). Retrieve samples at the designated time points for subsequent testing. Evaluate results based on the parameters specified in the detection procedure. |  |

**F.3.3 Determination of Storage Conditions and Shelf Life for Intermediate Samples**

The longest storage time of the validated experimental groups can be determined as the shelf life of the corresponding intermediate sample storage condition.

**Annex G**

**(Informative)**

**Examples for marker gene of residual PSCs detection**

**Table 3:** Representatives of reported marker gene for residual PSCs detection

| **Marker gene** | **Cell type** | **Reference** |
| --- | --- | --- |
| LIN28A | cardiomyocytes | ^1^Highly sensitive droplet digital PCR method for detection of residual undifferentiated cells in cardiomyocytes derived from human pluripotent stem cells，*Regen Ther.*2015 |
| miR-489-3p, miR-1/133a-3p, let-7c-5p | cardiomyocytes | ^2^Seamless and non-destructive monitoring of extracellular microRNAs duringcardiac differentiation from human pluripotent stem cells，Stem Cell Reports.2023 |
| ESRG , ZSCAN10 | cardiomyocytes, neural stem cells and hepatocytes | ^3^Universal Markers for hiPSCs Residue Detection，Front Biosci.2022 |
| ESRG, CNMD, SFRP2 | hepatocyte，pancreatic cell，neural crest cells and neural stem cells | ^4^Robust detection of undifferentiated iPSC among differentiated cells, Sci Rep.2020 |
| LIN28A, IDO1, CNMD | myotubes, myoblasts, neurons, hepatocytes,  cardiomyocytes, macrophages and monocytes | ^5^Identification of marker genes to monitor residual iPSCs in iPSC-derived products, Cytotherapy.2023 |
| POU5F1, LIN28,  TRA-2–49 | dopaminergic progenitor cells | ^6^Pre-clinical study of induced pluripotent stem cell-derived dopaminergic progenitor cells for Parkinson’s disease，Nat Commun.2020 |
| LNCPRESS2, LINC00678, LOC105370482 | islets | ^7^Estimating residual undifferentiated cells in human chemically induced pluripotent stem cell derived islets using lncRNA as biomarkers，Sci Rep.2020 |
| miR-302a-5p,   miR-302c-3p,  miR-302d-5p | natural killer cell | ^8^Development of an induced pluripotent stem cell specific microRNA assay for detection of residual undifferentiated cells in natural killer cell therapy products，Cytotherapy.2022 |
| LIN28A | Retinal pigment epithelial cell | ^9^Highly Sensitive In Vitro Methods for Detection of Residual Undifferentiated Cells in Retinal Pigment Epithelial Cells Derived from Human iPS Cells，PLoS One.2012 |

**Annex H**

**(Informative)**

**Study Protocol for Determination the Limit of Detection (LOD)**

**H.1 General**

To determine the LOD for residual PSC detection procedure, test gradient spiked samples (gradient numbers of PSCs added to a fixed number of negative control cells). Identify the minimum spiking level that can be consistently detected and shows significantly higher expression levels compared to pure negative control cells. This minimum spiking ratio is defined as the LOD for the corresponding detection procedure.

**H.2 Example of Experimental Design and Acceptance Criteria**

**H.2.1 Sample Preparation**

**H.2.1.1 Negative Control Cells and Gradient Spiked Samples:**
a. Prepare an adequate quantity of negative control cell samples according to experimental requirements. Adjust the cell suspension to an appropriate concentration based on cell counting.

b. Aliquot the suspension into 1.5 mL microcentrifuge tubes according to the cell number specified in the testing protocol (e.g., 2 × 10^6^ cells per sample). Use these aliquots to prepare negative control cell samples and gradient spiked samples as described below.

c. **Negative Control Cell Samples:** Centrifuge the aliquots from step b, discard the supernatant, and obtain parallel cell pellet replicates.

d. **Gradient Spiked Samples:** Spike the aliquots from step b with a gradient of PSC numbers (e.g., 2, 20, 200, 2000 PSCs). Centrifuge, discard the supernatant, and obtain parallel cell pellet replicates.

**H.2.1.2 Positive Control Cells**

Prepare PSC samples with equal cell number of the negative control cell sample, Centrifuge, discard the supernatant, and obtain parallel cell pellet replicates.

**H.2.2 Detection Experiment and Replication Validation**

**H.2.2.**1 Perform stem cell residual detection on negative control cell samples, gradient spiked samples, and positive control samples using the established detection procedure. Include ≥10 technical replicates per sample. Analyze and record the detection results.

**H.2.2.2** Repeat the LOD experiment using at least three independent batches of negative control cells following the same experimental protocol.

**H.2.3 LOD determination Criteria**

**H.2.3.1 Prerequisites for LOD determination:**

Experimental data can be used for LOD determination only if the following conditions are met:

a. Negative control cell samples show no detectable amplification (no typical amplification curve) or Ct values ≥ 35 in qPCR detection.

b. Positive control samples show positive amplification with Ct values ≤ 25.

**H.2.3.2 LOD Confirmation:**

**Case A:** If all negative control cell samples show no detectable amplification (no typical amplification curve):

Compare test results and select the lowest spiking ratio that is consistently detected in every replicate across all batches as the LOD for the corresponding detection procedure.

**Case B:** If some negative control cell samples show amplification with Ct values ≥ 35:

Compare test results and select the lowest spiking ratio where:

a. Every replicate across all batches is consistently detected.

b. The ∆∆Ct between the lowest detectable spiked sample (lowest relative marker gene expression) and the highest-expressing negative control sample is ≤ -1.

**References**

1. Kuroda T, Yasuda S, Matsuyama S, et al. Highly sensitive droplet digital PCR method for detection of residual undifferentiated cells in cardiomyocytes derived from human pluripotent stem cells. *Regen Ther.* 2015;2:17-23.

2. Sekine O, Kanaami S, Masumoto K, et al. Seamless and non-destructive monitoring of extracellular microRNAs during cardiac differentiation from human pluripotent stem cells. *Stem Cell Reports.* 2023;18(10):1925-1939.

3. Shi H, Feng T, Wang R, Wu L, Gu Y. Universal Markers for hiPSCs Residue Detection. *Front Biosci (Landmark Ed).* 2022;27(8):239.

4. Sekine K, Tsuzuki S, Yasui R, et al. Robust detection of undifferentiated iPSC among differentiated cells. *Sci Rep.* 2020;10(1):10293.

5. Lemmens M, Perner J, Potgeter L, et al. Identification of marker genes to monitor residual iPSCs in iPSC-derived products. *Cytotherapy.* 2023;25(1):59-67.

6. Doi D, Magotani H, Kikuchi T, et al. Pre-clinical study of induced pluripotent stem cell-derived dopaminergic progenitor cells for Parkinson's disease. *Nat Commun.* 2020;11(1):3369.

7. Wu Y, Zhang Z, Wu S, Chen Z, Pu Y. Estimating residual undifferentiated cells in human chemically induced pluripotent stem cell derived islets using lncRNA as biomarkers. *Sci Rep.* 2023;13(1):16435.

8. Chung L, Cogburn LA, Sui L, Dashnau JL. Development of an induced pluripotent stem cell-specific microRNA assay for detection of residual undifferentiated cells in natural killer cell therapy products. *Cytotherapy.* 2022;24(7):733-741.

9. Kuroda T, Yasuda S, Kusakawa S, et al. Highly sensitive in vitro methods for detection of residual undifferentiated cells in retinal pigment epithelial cells derived from human iPS cells. *PLoS One.* 2012;7(5):e37342.
